# Multi-Metal Alloys as Catalysts for Fenton-like Oxidation: A Review

**DOI:** 10.3390/ma19061220

**Published:** 2026-03-19

**Authors:** Wenjun Sun, Bingbing Li, Wenqiang Dong, Qixing Xia

**Affiliations:** 1 Key Laboratory of Archaeological Exploration and Cultural Heritage Conservation Technology (Northwestern Polytechnical University), Ministry of Education, Xi’an 710129, China; 2 School of Life Science and Technology, Northwestern Polytechnical University, Xi’an 710129, China; 3 School of Public Policy and Administration, Northwestern Polytechnical University, Xi’an 710072, China; 4 Institute of Culture and Heritage, Northwestern Polytechnical University, Xi’an 710072, China

**Keywords:** Fenton-like oxidation, multi-metal alloys, catalytic mechanisms, radical pathways, synergistic effects

## Abstract

The persistent discharge of refractory toxic organic pollutants poses a severe threat to aquatic environmental safety, driving the urgent demand for high-efficiency water treatment technologies in environmental engineering. Fenton and Fenton-like oxidation processes have garnered extensive attention due to their robust oxidizing capacity and environmental benignity; however, traditional Fenton systems are constrained by inherent limitations, including a narrow applicable pH range, potential secondary pollution, and cumbersome catalyst recovery. To address these challenges, Fenton-like catalysts have evolved progressively from single-metal systems to multi-metal alloy configurations. This review systematically elaborates on the fundamental principles and technical bottlenecks of classical Fenton and Fenton-like reactions, while comprehensively summarizing the research progress of multi-metal alloy catalysts—encompassing binary alloys, multi-component alloys, and high-entropy alloys. Special emphasis is placed on dissecting the core mechanisms through which multi-metal alloys optimize redox cycles and enhance structural stability, leveraging intermetallic synergistic effects, unique electronic structures, and lattice distortion. Furthermore, this work synthesizes key performance enhancement strategies for such catalysts, including co-catalyst synergy, external field assistance, and supported composite modification. Ultimately, this review aims to provide a scientific foundation and technical reference for the rational design, development, and engineering application of high-performance Fenton-like catalysts in sustainable wastewater remediation.

## 1. Introduction

With the continuous advancement of industrialization and the persistent discharge of various emerging contaminants, the concentration of recalcitrant and toxic organic pollutants in industrial wastewater and domestic sewage has risen significantly. This poses a severe threat to aquatic ecological balance and human health. These pollutants are typically characterized by stable molecular structures, high resistance to degradation, and a tendency for environmental bioaccumulation, making it difficult for traditional water treatment technologies to achieve efficient removal [1]. Driven by the global consensus on sustainable development and ecological protection, the development of high-efficiency, green, and cost-effective water pollution control technologies has emerged as a critical research priority in the field of environmental protection [1,2].

Advanced Oxidation Processes (AOPs) have become a key pathway for the degradation of recalcitrant organic pollutants by generating highly reactive oxidative species [3]. Among these, Fenton and Fenton-like technologies have garnered significant attention due to their rapid reaction kinetics, potent oxidative capacity, and environmental friendliness [4]. The classical Fenton reaction utilizes Fe^2+^ to activate H_2_O_2_, generating hydroxyl radicals (·OH). With an oxidation potential of 2.8 V—second only to fluorine—OH can non-selectively mineralize the majority of organic pollutants [5]. However, the practical application of this technology is still hindered by distinct limitations, including the high storage and transportation costs and low safety of H_2_O_2_, the generation of iron sludge causing secondary pollution, the requirement for strictly acidic operating conditions necessitating subsequent neutralization steps, and stringent requirements for equipment corrosion resistance.

To overcome these bottlenecks, researchers have developed enhanced Fenton-like systems by introducing complexing agents, metal ion doping, and external field coupling. These systems not only broaden the applicable pH range and improve catalytic efficiency but also reduce reagent consumption; the core of these advancements lies in the development of high-performance catalysts [6]. Currently, Fenton-like catalysts have evolved from monometallic systems (e.g., Fe-based, Cu-based) toward multi-metal alloy systems [7]. Leveraging intermetallic synergistic effects, unique electronic structures, and superior stability, multi-component alloys effectively address the issues of particle aggregation, active site leaching, and poor cyclic stability inherent in monometallic catalysts [8]. Furthermore, they can efficiently activate oxidants such as hydrogen peroxide and persulfates, further expanding the scope of technical applications.

This paper systematically elucidates the fundamental principles and technical limitations of classical Fenton and Fenton-like reactions and reviews the research progress of monometallic Fenton-like catalytic materials [9]. Particular emphasis is placed on analyzing the synthesis methods, catalytic performance, and synergistic mechanisms of multi-metal alloy catalysts, including binary alloys, multi-component alloys, and high-entropy alloys (HEAs) [10]. Additionally, performance enhancement strategies—such as co-catalyst synergy, external field assistance, and supported composite modification—are introduced [11]. This work aims to provide a scientific foundation and technical reference for the design and development of high-performance Fenton-like catalysts and their practical application in aquatic environmental remediation.

## 2. Fenton/Fenton-like Reactions and Characteristics of Different Metal Species

### 2.1. Classical Fenton Reaction

In 1894, the British chemist H.J.H. Fenton first reported the classical Fenton reaction, establishing a pivotal branch of Advanced Oxidation Processes (AOPs) [12]. The core mechanism involves the catalytic decomposition of H_2_O_2_ by Fe^2+^ to generate highly reactive hydroxyl radicals ·OH), thereby achieving the efficient oxidative degradation of organic pollutants. H_2_O_2_ itself is considered an environmentally friendly “green” oxidant, as its decomposition products are limited to water and oxygen [13,14]. The ·OH radical possesses an extraordinary oxidative capacity, with a standard oxidation potential as high as 2.8 V—significantly higher than the 1.76 V of H_2_O_2_—making it the critical active species for pollutant degradation in Fenton systems [11]. Due to its rapid reaction kinetics, operational simplicity, and the absence of toxic byproducts, this technology has been widely implemented in treating recalcitrant organic pollutants within industrial and municipal wastewater [15,16,17].

However, the practical application of the classical Fenton process faces several constraints: the high cost and safety risks associated with the storage and transport of H_2_O_2_; the inevitable generation of iron sludge, leading to secondary pollution; and the requirement for strictly acidic conditions, which necessitates subsequent neutralization [18,19]. Furthermore, the high demand for corrosion-resistant equipment further restricts its large-scale engineering application [16].

### 2.2. Fenton-like Reactions

To circumvent the limitations of the classical Fenton reaction and enhance its oxidative efficiency, extensive modification research has been conducted in recent years focusing on reaction mechanisms, operational conditions, and catalyst systems. This has led to the development of a series of novel AOPs derived from the classical Fenton process, collectively termed “Fenton-like” reactions [20]. By implementing strategies such as the introduction of chelating agents, regulation of active species morphology, doping with heterogeneous metal ions, or coupling with external energy fields, Fenton-like technologies have successfully broadened the applicable pH range, significantly improved catalytic efficiency, and reduced reagent consumption [21]. Compared to classical Fenton reactions, Fenton-like systems utilize a wider array of raw materials and demonstrate greater potential for optimization in terms of catalytic activity and structural stability [22].

Fenton-like technologies are primarily categorized into homogeneous and heterogeneous systems, with most transition metals serving as catalysts in heterogeneous Fenton reactions [23,24]. Unlike homogeneous systems, heterogeneous Fenton reactions occur at the liquid–solid interface through several key steps: the diffusion of H_2_O_2_ within the system followed by its selective adsorption onto the catalyst surface; the in situ generation of ·OH at the catalyst surface, where a portion of the radicals diffuse into the bulk liquid phase to react with organic matter, while others react directly with adsorbed organic molecules on the surface. Simultaneously, the process involves the mass transfer of organic pollutants from the liquid phase to the catalyst surface, as well as the desorption and subsequent decomposition of degradation intermediates. While the reaction mechanism of heterogeneous Fenton technology is more complex and influenced by a broader range of factors, it offers significantly more latitude for precise regulation and systematic optimization [25]. To further quantify these chemical processes, the key stoichiometric reactions and the corresponding activation energy ranges for both single-metal and multi-metal alloy systems are summarized in Table 1.

### 2.3. Characteristics of Different Metal Species in Fenton-like Reactions

Multivalent metals (e.g., Fe, Cu, Co, Mn) leverage their redox properties to facilitate valence cycling through electron transfer with H_2_O_2_, simultaneously inducing the generation of reactive oxygen species (ROS) [35,36,37]. The mechanism of valence cycling and ROS generation is as follows: the lower-valence metal acts as a nucleophile, attacking the O-O bond of H_2_O_2_ to generate hydroxyl radicals via a single-electron reduction while being oxidized to a higher valence state [34]; subsequently, the higher-valence metal acts as an electrophile, abstracting a hydrogen atom from H_2_O_2_ to decompose it into superoxide radicals, thereby restoring the metal to its lower-valence state to complete the catalytic cycle [32].

Fenton-like reaction pathways are generally classified into radical and non-radical pathways. The radical pathway is dominated by ·OH and SO^4−^; iron- and cobalt-based systems primarily degrade pollutants through this route, which offers extremely high oxidation potentials but is susceptible to quenching by inorganic anions (e.g., Cl^−^, HCO_3−_) present in the water matrix. The non-radical pathway involves high-valent metal-oxo complexes (e.g., Fe (IV)=O, Cu (III)) or singlet oxygen [38]. Certain metal species, such as Cu or Mn in specific coordination environments, tend to form transient high-valent metal intermediates. Compared to radicals, this pathway exhibits higher selectivity and stronger anti-interference capabilities in complex water matrices [39]. For instance, in certain heterogeneous catalytic systems, surface-bound active sites can undergo direct two-electron transfer with organic pollutants without releasing free radicals [34].

Multi-metal alloys offer significant synergistic advantages that effectively overcome the limitations of monometallic catalysts. Traditional iron-based Fenton reactions are restricted to a narrow pH range of 2–3, as iron ions tend to undergo hydrolysis and precipitation at higher pH levels. However, the introduction of metals such as Cu or Co to form alloys can significantly enhance reaction rates under neutral conditions [40]-Cu, for example, demonstrates excellent solubility and catalytic activity within pH 3–5 or even wider ranges [40]. Alloying can regulate the binding energy of metal atoms through lattice distortion or the formation of surface passivation layers, thereby inhibiting metal leaching during the catalytic process. Furthermore, the synergistic support of multiple components effectively mitigates the aggregation of active sites during cycling, maintaining the long-term mechanical strength and chemical stability of the catalyst [41]. In Fe-Cu or Fe-Co alloys, the electronic coupling between different metal redox pairs can significantly accelerate the rate-limiting steps (Table 2).

## 3. Classification and Progress of Multi-Metal Alloy Catalytic Materials

### 3.1. Single-Metal Catalytic Materials

The monometallic catalytic materials described in this section refer to Fenton-like catalysts featuring a single transition metal (Fe, Cu or Co) as the core active component. This category encompasses pure metals, metal oxides, and simple supported materials, distinguishing them from the multi-component systems of multi-metallic alloys discussed in subsequent sections.

#### 3.1.1. Iron-Based Fenton-like Catalysts

Iron, as an abundant element in the Earth’s crust, has been extensively utilized in the field of environmental remediation. It exists in three primary oxidation states, zero-valent iron (ZVI), ferrous ions (Fe^2+^), and ferric ions (Fe^3+^), all of which can induce Fenton or Fenton-like processes [39,45,46]. Among these, the valence state transformation of Fe^2+^/Fe^3+^ is particularly critical [47,48]. This transformation is thermodynamically feasible, primarily because the standard reduction potentials of Fe^2+^/Fe^0^ (−0.44 V) and Fe^2+^/Fe^3+^ (+0.776 V) are both lower than that of H_2_O_2_/·OH (+1.44 V), while the Fe^3+^/Fe^2+^ potential is higher than that of O_2_/H_2_O_2_ (+0.695 V), thereby providing the driving force for the iron cycle [33,49,50].

Traditional Fe-based monometallic catalysts commonly exhibit morphologies such as nanoparticles, nanorods, and nanosheets. Frequently utilized supports for these catalysts include silica (SiO_2_), alumina (Al_2_O_3_), SBA-15, and activated carbon. However, unsupported Fe-based catalysts are highly susceptible to aggregation and the leaching of active sites [51].

In acidic ZVI/H_2_O_2_ systems, Fe^0^ reacts with H^+^ and H_2_O_2_ to generate Fe^2+^. Simultaneously, the Fe^3+^ produced by the oxidation of Fe^2+^ can be rapidly reduced back to Fe^2+^ by Fe^0^ [34]. This mechanism effectively accelerates the ≡Fe^3+^/Fe^2+^ redox cycle and promotes the generation of reactive oxygen species (ROS) [38]. However, in traditional Fenton systems, the direct reduction of Fe^3+^ to Fe^2+^ is thermodynamically unfavorable for spontaneous occurrence, as the potential of ·OOH/H_2_O_2_ (+1.14 V) is higher than that of Fe^3+^/Fe^2+^. To address this, Zakharov et al. [52] discovered through quantum chemical calculations that the presence of sulfate ligands in the first coordination sphere of iron–aqua complexes allows for the spontaneous decomposition of H_2_O_2_. This alters the thermodynamic reaction pathway and facilitates the Fe^3+^/Fe^2+^ cycle [53,54,55].

The Fe^3+^/Fe^2+^ cycle is regulated by various factors, including pH, ligands, and light irradiation. Typically, this cycle proceeds efficiently only under specific conditions: in neutral environments, Fe^3+^ is prone to hydrolysis, leading to decreased reactivity with H_2_O_2_ [49]. However, under the synergistic effect of ligands and light, certain Fe^3+^ hydroxy or organic complexes can regenerate Fe^2+^ via Ligand-to-Metal Charge Transfer (LMCT) processes when exposed to UVA or visible light. Furthermore, under appropriate ligand and pH conditions, high-valent iron-oxo intermediates (Fe(IV) = O) can also participate in the iron cycle and radical generation. At pH > 3, these intermediates primarily exist as FeIV(OH)_2_^2+^ and FeIV(OH)_3_^+^. Under neutral pH conditions, [(H_2_O)_3_FeIV(OH)_3_]^+^ is the predominant species, while [(H_2_O)_3_FeIV(OH)_4_] may also coexist [56].

#### 3.1.2. Copper-Based Fenton-like Catalysts

Copper exists in three oxidation states, metallic copper (Cu^0^), cuprous ions (Cu^+^), and cupric ions (Cu^2+^), all of which can induce the decomposition of hydrogen peroxide H_2_O_2_) to generate reactive oxygen species (ROS) [57]. In copper-catalyzed Fenton-like processes, the redox cycle primarily occurs between Cu^+^ and Cu^2+^, a mechanism analogous to the Fe^2+^/Fe^3+^ redox cycle observed in traditional Fe/H_2_O_2_ systems [27,58,59,60].

Compared to traditional iron-based Fenton processes, copper-based Fenton-like systems exhibit a broader applicable pH window [61]. This advantage primarily stems from the higher solubility of Cu^2+^ in aqueous solutions, enabling it to maintain catalytic activity under near-neutral or even weakly alkaline conditions. Furthermore, Cu^2+^ readily forms complexes with organic pollutants and their oxidative intermediates [62]. These complexes can be efficiently decomposed by the hydroxyl radicals generated within the system, thereby promoting the deep mineralization of organic contaminants. However, this system faces certain challenges in practical applications: because Cu^+^ is highly susceptible to oxidation by dissolved oxygen (O_2_), the regeneration efficiency of active Cu^+^ during the catalytic cycle is hindered [63]. Consequently, a higher dosage of H_2_O_2_ is often required to sustain reaction efficiency, necessitating further optimization of its economic feasibility relative to iron-based systems [20,64,65].

#### 3.1.3. Cobalt-Based Fenton-like Catalysts

The catalytic activity in cobalt-based Fenton-like systems is highly dependent on the stability of the Co^3+^/Co^2+^ redox couple. The standard reduction potential of this couple is +1.92 V, significantly higher than that of the O_2_/H_2_O couple (+1.23 V), indicating that free Co^3+^ is extremely prone to disproportionation or reduction in aqueous solutions, making it difficult to exist stably [66,67,68]. To maintain an effective catalytic cycle, two strategies are commonly employed in practice: the first involves the use of Co^2+^ complexes coordinated with ligands (such as water, ammonia, bicarbonate, or ascorbic acid) to stabilize the metal center through coordination; the second involves the construction of heterogeneous Co^2+^ catalysts to restrict the leaching and transformation of cobalt ions using solid-phase supports [69,70,71].

In ligand-modified homogeneous systems, the ligand first forms a stable complex with Co^2+^, which subsequently reacts with H_2_O_2_ to generate highly reactive cobalt-peroxo intermediates [69,72]. These intermediates can further decompose via homolytic or heterolytic cleavage pathways to produce hydroxyl radicals (·OH) or directly undergo electron transfer with organic pollutants to form organic radicals. It is important to note that the generation mechanism of ROS is not singular; the specific pathway strongly depends on the nature of the ligands and the reaction environment. For instance, in the ternary Co^2+^/HCO_3_^−^/H_2_O_2_ system [40], the reduction of Co^3+^ to Co^2+^ is accompanied by the generation of superoxide anion radicals. This species not only participates in radical chain reactions but also contributes significantly to the oxidative decolorization of dyes [35]. Therefore, through the rational design of ligand structures and the regulation of reaction conditions, directed control over the ROS generation pathways can be achieved, thereby optimizing the oxidative performance of the catalytic system [28,73,74].

### 3.2. Binary Alloy Systems

#### 3.2.1. Fe–Cu Alloy System

**Figure 1:**
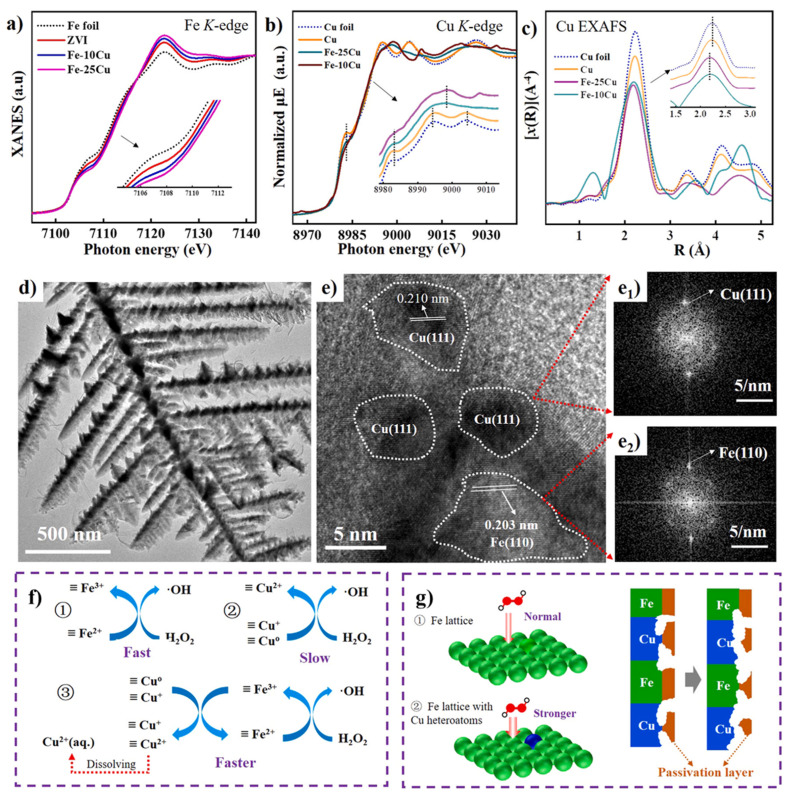
(**a**) Fe K–edge XANES spectra of ZVI, Fe–10Cu, Fe–25Cu, and Fe foil [78]; (**b**) Cu K–edge XANES spectra and [78]; (**c**) EXAFS spectra of 100Cu, Fe–10Cu, Fe–25Cu, and Cu foil [78]; (**d**) TEM and (**e**) HRTEM images of Fe–25Cu, along with (**e_1_**,**e_2_**) FFT patterns corresponding to different regions in the HRTEM image [78]; (**f**) acceleration of the Fe^2+^/Fe^3+^ transformation by copper species [78]; (**g**) inhibition of the passivation layer formation on the Fe-25Cu surface by independent copper clusters [78].

**Figure 2:**
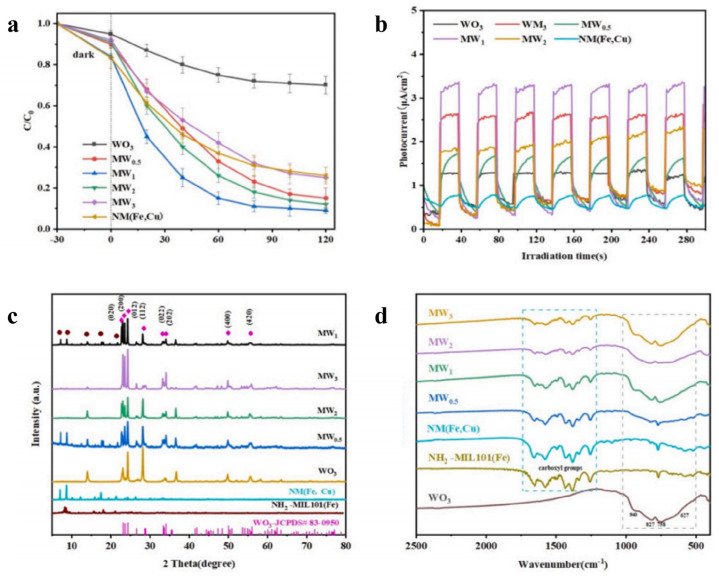
(**a**) Photocatalytic rate of LEV under visible light irradiation on as-prepared samples; (**b**) Transient photocurrent response (**c**) The XRD patterns and (**d**) the FTIR spectra of the WO_3_, NM (Fe, Cu), NH_2_–MIL101(Fe), and NM (Fe, Cu) [44].

The Fe–Cu alloy system has emerged as one of the most promising bimetallic catalysts in Fenton-like oxidation processes, owing to the significant synergistic effects between the iron (Fe) and copper (Cu) components [6,36,75,76]. The introduction of copper effectively overcomes a critical bottleneck in traditional Fenton reactions [4]: the sluggish reduction cycle of Fe^3+^/Fe^2+^. For instance, research by Xia et al. [77] confirmed (Figure 1) that Cu heteroatoms and independent Cu clusters within the Fe–Cu alloy lattice facilitate efficient interfacial electron transfer, thereby significantly accelerating the regeneration of active Fe^2+^ sites. As illustrated in the Fe K-edge XANES spectra (Figure 1a), the absorption edges of Fe-10Cu and Fe-25Cu exhibit a visible shift toward lower-energy regions compared to pristine ZVI. This shift indicates that the incorporation of Cu reduces the average oxidation state of Fe, thereby increasing the content of Fe^2+^/Fe^3+^ active sites—where Fe^2+^ serves as the core site for H_2_O_2_ activation to generate ·OH. Furthermore, the decreased peak intensity in the R-space of Cu EXAFS spectra (Figure 2c) for the Fe-Cu alloys suggests a distortion in the Cu coordination environment and a contraction in bond length. Such lattice distortion optimizes the electron density on the catalyst surface and accelerates interfacial electron transfer, which subsequently enhances the Fe^2+^/Fe^3+^ cycling rate and accounts for the superior catalytic performance of Fe-Cu alloys over pure Fe-based catalysts. The typical effective temperature range for the Fe-Cu alloy-catalyzed Fenton-like reaction is 25–50 °C, with an apparent activation energy (Ea) of 22–38 kJ/mol, which is significantly lower than that of pure Fe (45–65 kJ/mol; specifically 53 kJ/mol in previous reports). According to the Arrhenius equation, the introduction of Cu heteroatoms into the Fe-Cu alloy leads to a decrease in the average oxidation state of Fe (as evidenced by the red shift in XANES spectra). Furthermore, the lattice distortion optimizes the surface electron density, thereby substantially lowering the energy barrier for H_2_O_2_ activation. Consequently, the reaction rate constant at 25 °C is 2.5–3 times higher than that of pure Fe, maintaining a Rhodamine B degradation efficiency of over 95%.

Electron paramagnetic resonance (ESR/EPR) analysis further elucidates the mechanism of this synergy: compared to monometallic iron-based catalysts, the Fe–Cu bimetallic system is capable of generating a higher abundance of hydroxyl radicals (·OH) and superoxide radicals.

Furthermore, Fe–Cu alloy catalysts exhibit excellent structural stability and recyclability [79]. The presence of Cu helps inhibit the formation of an iron oxide passivation layer on the catalyst surface, thereby maintaining the effective exposure of active sites during long-term operation [80]. To enhance catalytic efficiency, researchers have further explored dispersing and supporting Fe–Cu active components on various carrier materials. Lu et al. [43] synthesized a series of FexCu_5_-x/HZSM-5 catalysts and discovered that the interaction between Fe and Cu on the HZSM-5 surface provides a favorable environment for the efficient degradation of Rhodamine B. Similarly, encapsulating Fe–Cu bimetallic oxides within the channels of ZSM-5 zeolites can significantly enhance the heterogeneous Fenton-like degradation of methylene blue by increasing the specific surface area and promoting the synergistic redox cycles of Cu^2+^/Cu^+^ and Fe^3+^/Fe^2+^.

In more advanced configurations, Fe–Cu bimetallic catalysts have been integrated with photoactive materials to construct high-efficiency light-driven Fenton-like systems. Liu et al. [44] reported that the construction of a Z-scheme heterojunction in NH_2_–MIL101(Fe,Cu)/WO_3_ significantly promoted the separation of photogenerated charge carriers, achieving a 90% degradation rate of levofloxacin under visible light irradiation (Figure 2). Additionally, utilizing biochar as a support for Cu-Fe bimetals provides a sustainable strategy for catalyst system construction, where nitrogen doping within the biochar facilitates the formation of S-scheme heterojunctions, thereby comprehensively improving the catalytic removal performance for pollutants such as sulfamethoxazole.

#### 3.2.2. Fe–Ni Alloy System

Iron–nickel (Fe–Ni) bimetallic alloys, as a significant class of heterogeneous catalysts, have demonstrated substantial potential in Fenton-like processes [81]. This is primarily attributed to the prominent electronic interactions between Fe and Ni, which not only facilitate the redistribution of electron density on the catalyst surface but also effectively accelerate the Fe^3+^/Fe^2+^ redox cycle [46,82,83], thereby enhancing the regeneration efficiency of active sites. In these systems, iron typically serves as the primary active center for H_2_O_2_ activation, while the introduction of nickel plays a crucial role in electronic regulation, improving overall catalytic performance through synergistic electron transfer [84].

Recent studies indicate that Fe–Ni alloys exhibit excellent versatility across various Fenton-like systems, including dark Fenton, photo-Fenton, and electro-Fenton processes. For instance, Keselytė et al. [42] recovered Ni–Fe bimetallic catalysts from semiconductor wastewater using a fluidized-bed crystallization reactor and applied them to the degradation of Reactive Black 5 dye. The catalyst showed superior catalytic performance under heterogeneous Fenton-like conditions: at an optimal catalyst dosage (4 g/L) and H_2_O_2_ concentration (8.83 mM/L), efficient mineralization of the azo dye was achieved. The research demonstrated that the presence of nickel significantly facilitated the reaction process and maintained exceptionally high removal efficiency over a wide pH range.

**Figure 3:**
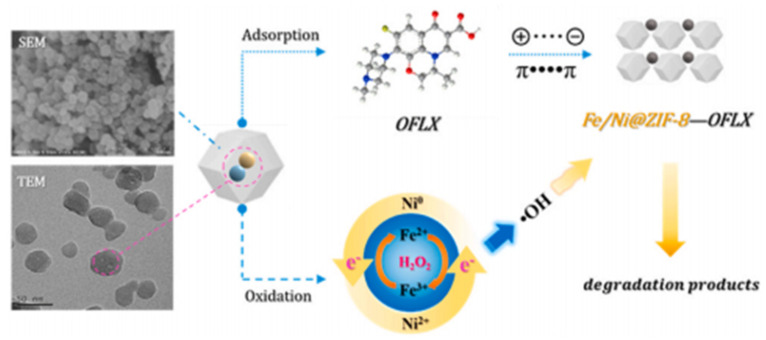
Adsorption by ZIF−8 and the Fenton-like oxidation process induced by Fe/Ni alloys within the pores [81].

To further improve the stability and specific surface area of Fe-Ni catalysts, the metal–organic framework (MOF) template method has been widely adopted. Zhang et al. [81] successfully constructed a ZIF-8@Fe/Ni composite catalyst by embedding bimetallic Fe/Ni nanoparticles into a ZIF-8 framework (Figure 3). This system integrates the high adsorption capacity of ZIF-8 with the catalytic activity of the Fe/Ni alloy, establishing a synergistic “adsorption–oxidation” mechanism that exhibits superior performance in the removal of ofloxacin. The synergy between Fe and Ni within the ZIF-8 matrix significantly promotes the generation of hydroxyl radicals, leading to a pollutant removal rate exceeding 90%.

**Figure 4:**
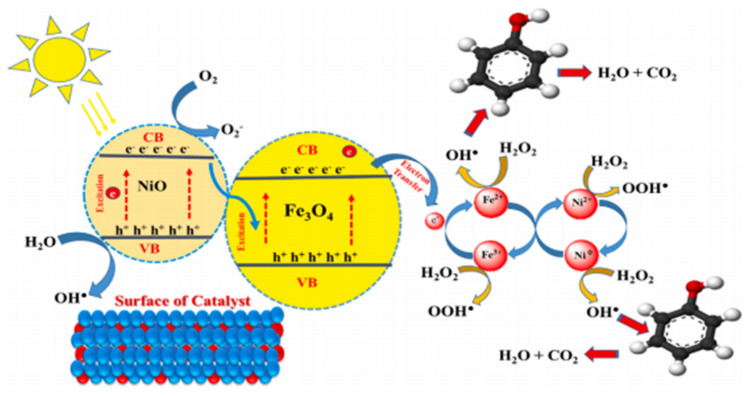
Proposed mechanism for the photo−Fenton-like degradation of phenol [85].

The synergy between Fe-Ni alloys and light irradiation offers significant advantages in accelerating the rate-limiting steps of the Fenton reaction. Soomro et al. [85] developed an iron–nickel bimetallic nanoalloy for the photo-Fenton degradation of phenol. The alloy structure possesses a high specific surface area, which enhances its catalytic activity. Under UV-visible light irradiation, the Ni component effectively promotes the reduction of Fe^3+^ to Fe^2+^, thereby maintaining a high concentration of active Fe^2+^ and successfully overcoming the issue of sluggish Fe^3+^/Fe^2+^ cycling inherent in traditional iron-based catalysts (Figure 4).

**Figure 5:**
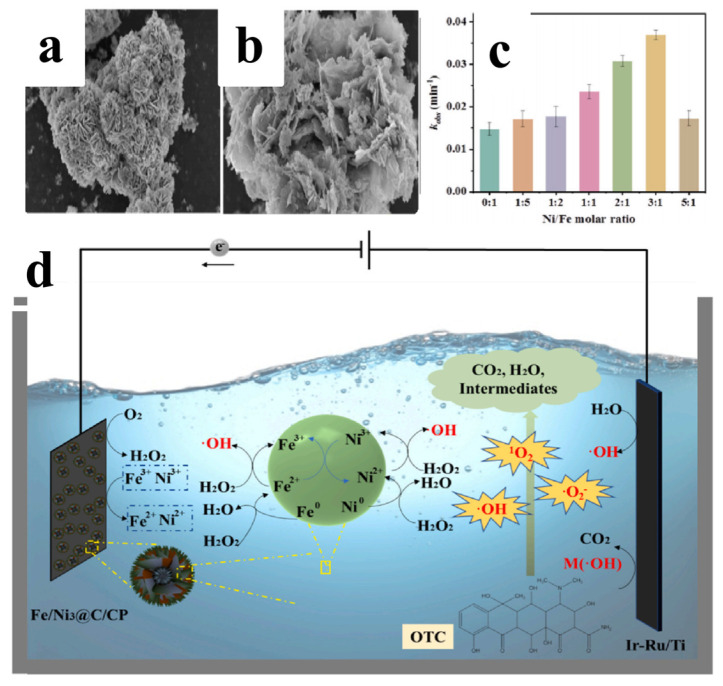
(**a**,**b**) SEM images of FeNi_3_@C [9]; (**c**) kinetic constants of OTC degradation by catalysts with different Fe/Ni molar ratios [9]; (**d**) Proposed reaction mechanism for oxytetracycline (OTC) degradation via the heterogeneous electro−Fenton system using FeNi_3_@C/CP [9].

In the field of electro-Fenton (EF) oxidation, Fe–Ni alloys have been designed as high-performance cathodes to simultaneously achieve the generation and activation of H_2_O_2_. Liu et al. [9]. prepared MOF-derived Fe/Ni@C marigold-like nanosheets and utilized them as a heterogeneous EF cathode for the degradation of oxytetracycline (Figure 5a,b). This marigold-like structure provides abundant active sites, while the Fe/Ni bimetallic centers act as bifunctional catalysts, capable of both efficiently catalyzing the two-electron oxygen reduction reaction (ORR) to produce H_2_O_2_ and subsequently converting it into ·OH (Figure 5d). The researchers specifically emphasized that Ni-induced electronic regulation lowers the energy barrier for the formation of key intermediates, significantly enhancing the overall EF efficiency compared to pure Fe@C catalysts (Figure 5c).

#### 3.2.3. Cu–Ni Alloy System

The Cu–Ni alloy system has garnered extensive attention in the field of Fenton-like oxidation due to the significant synergistic effects between the copper (Cu) and nickel (Ni) components, with catalytic performance markedly superior to that of the corresponding monometallic materials [86,87,88,89,90]. Within this bimetallic structure, the introduction of Ni effectively facilitates the valence state cycling of Cu species, thereby enhancing the efficiency of hydrogen peroxide (H_2_O_2_) decomposition into reactive oxygen species (ROS) [91,92,93].

**Figure 6:**
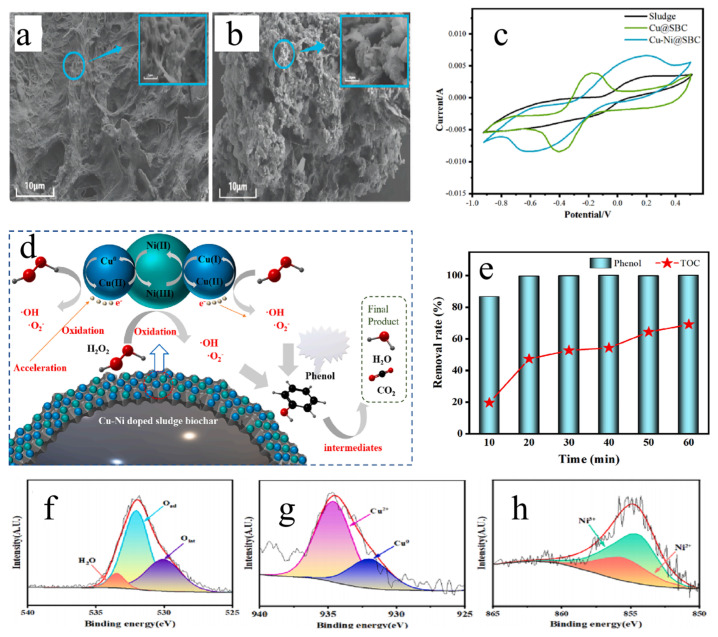
(**a**,**b**) SEM images of (**a**) raw sludge and (**b**) Cu−Ni@SBC [86]; (**c**) CV curves of Raw sludge, Cu@SBC and Cu−Ni@SBC materials [86]; (**d**) Reaction mechanism of phenol degradation in Cu−Ni@SBC + H_2_O_2_ system [86]; (**e**) Removal efficiency of phenol and TOC in solution; (**f**–**h**) XPS peak-fitting spectrum of Cu and Ni elements in Cu−Ni@SBC [86].

For instance, Liu et al. [86] developed a Cu–Ni bimetal-doped sewage sludge biochar catalyst (Cu–Ni@SBC). The synergistic redox cycles between Cu0/Cu^2+^ and Ni^3+^/Ni^2+^ significantly promoted the generation of ·OH, intensifying the degradation process. By activating H_2_O_2_ to degrade aqueous phenol, the study systematically elucidated the mechanisms of synergistically enhanced electron transfer and radical generation, while also analyzing the causes of decreased degradation efficiency due to radical quenching (Figure 6).

**Figure 7:**
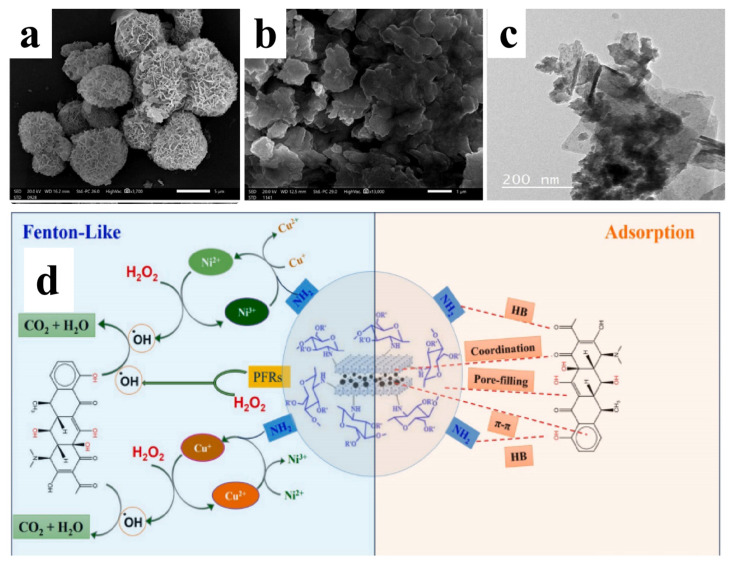
SEM images of (**a**) dandelion-like-structure Cu-Ni LDH, (**b**) Cu-Ni LDH/BC/AmCS, (**c**) TEM of Cu-Ni LDH/BC/AmCS; (**d**) Schematic illustration of the Fenton-like degradation mechanism of doxycycline (DOX) by the Cu–NiLDH/BC/AmCS composite.

Furthermore, compositing Cu-Ni alloys with carbon-based supports can further improve Fenton-like performance. Eltaweil et al. [7] synthesized a dandelion-like ternary composite catalyst consisting of Cu-Ni layered double hydroxides supported on biochar/aminated chitosan (Cu-Ni LDH/BC/AmCS). This architecture not only synergistically improves H_2_O_2_ activation efficiency through the electron transfer between the Cu/Ni bimetallic cycle and the carbonaceous support but also inhibits metal agglomeration via the LDH–carbon–polymer composite system. Consequently, it demonstrated superior performance in the Fenton-like degradation of doxycycline (Figure 7).

#### 3.2.4. Fe–Co Alloy System

Fe–Co binary alloy has emerged as a highly promising heterogeneous Fenton-like catalyst due to the strong synergistic electronic interaction between Fe and Co components. This synergy not only optimizes the surface electron density distribution of the catalyst but also constructs a mutually promoted redox cycle between Fe^3+^/Fe^2+^, which effectively overcomes the rate-limiting step of Fe^3+^ reduction in traditional iron-based Fenton systems and significantly enhances the activation efficiency of oxidants such as H_2_O_2_ or peroxymonosulfate (PMS) [94,95,96]. In the Fe–Co alloy structure, Fe typically serves as the primary active center for oxidant activation, while Co acts as an electronic regulator to accelerate interfacial electron transfer, thereby improving the overall catalytic performance through synergistic effects [26,97,98].

**Figure 8:**
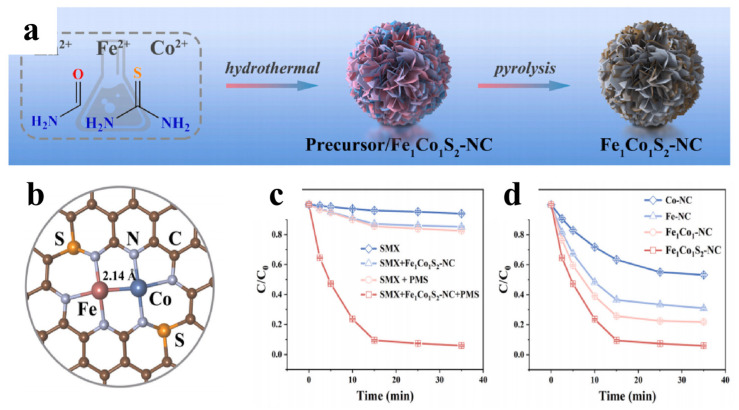
(**a**) Schematic representation of the catalyst synthesis [99]; (**b**) atomic structure modeling; (**c**) Degradation–time curves of SMX under different catalytic systems [99]; (**d**) Degradation–time curves [99].

Cheng et al. [99] developed a sulfur-doped Fe/Co dual-atom catalyst (Figure 8). The electronic structure optimization of active sites by S doping coupled with the synergistic effect of Fe/Co dual-atom sites significantly promoted the generation of the high-valent iron-oxygen-cobalt bridged complex, intensifying the degradation process of organic pollutants in water. By activating peroxymonosulfate (PMS) to degrade aqueous sulfamethoxazole (SMX) and other typical pollutants, the study systematically elucidated the mechanisms of electronic structure engineering facilitating electron transfer from metallic active sites to oxygen atoms, lowering the formation energy barrier of high-valent metal-oxo (HVMO) species and enhancing their oxidation capacity, while also clarifying the reason that traditional radical species (•OH, SO_4_•^−^, ^1^O_2_) had negligible contributions to pollutant removal (Figure 8).

#### 3.2.5. Cu–Co Alloy System

The copper–cobalt (Cu–Co) bimetallic system has attracted widespread research interest due to the unique synergistic effects arising from the interaction between Cu and Co redox couples [35]. The introduction of copper into cobalt-based catalysts typically promotes the regeneration of low-valence metal species (Co^2+^ and Cu^+^) through interfacial electron transfer, thereby accelerating the rate-determining step of Fenton-like reactions. This interaction effectively mitigates the accumulation of high-valence species, maintaining high catalytic activity and improving oxidant utilization efficiency [62].

Recent progress has highlighted the efficacy of metal–organic framework (MOF)-derived Cu-Co structures in heterogeneous Fenton systems. Zhou et al. [35] successfully synthesized Cu–Co binary MOF nanosheets via a one-pot method for the efficient degradation of norfloxacin. The two-dimensional structure of the MOF nanosheets provides a high specific surface area and exposes abundant active sites for H_2_O_2_ activation. The researchers observed that the coexistence of Cu and Co species within the MOF framework significantly enhanced catalytic efficiency compared to their monometallic counterparts. This enhancement is attributed to a dual-active-site mechanism, where the Co(II)/Co(III) and Cu(I)/Cu(II) cycles work synergistically to activate H_2_O_2_, leading to the rapid generation of hydroxyl radicals (·OH).

**Figure 9:**
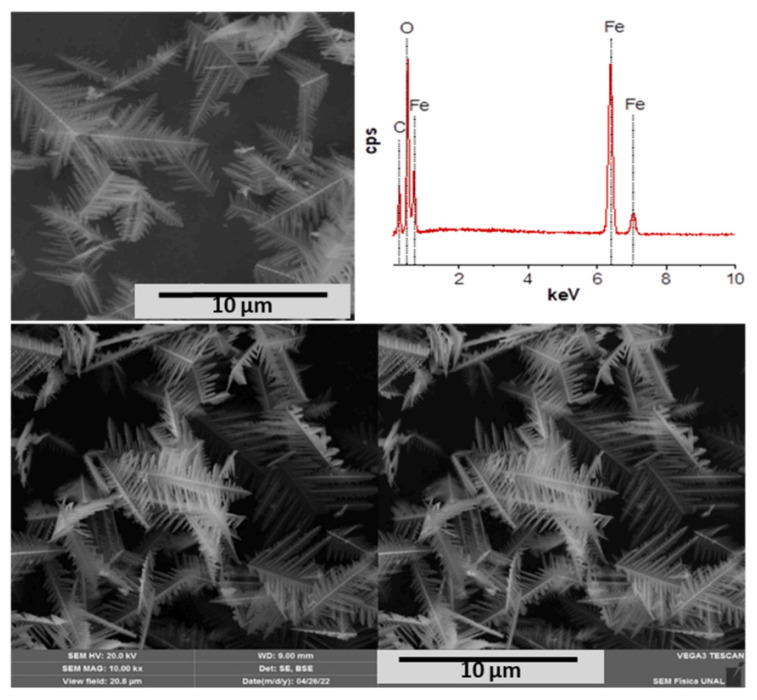
Representative SEM micrographs and EDX analysis of the catalytic support (MB-H:α-Fe_2_O_3_) [14].

**Figure 10:**
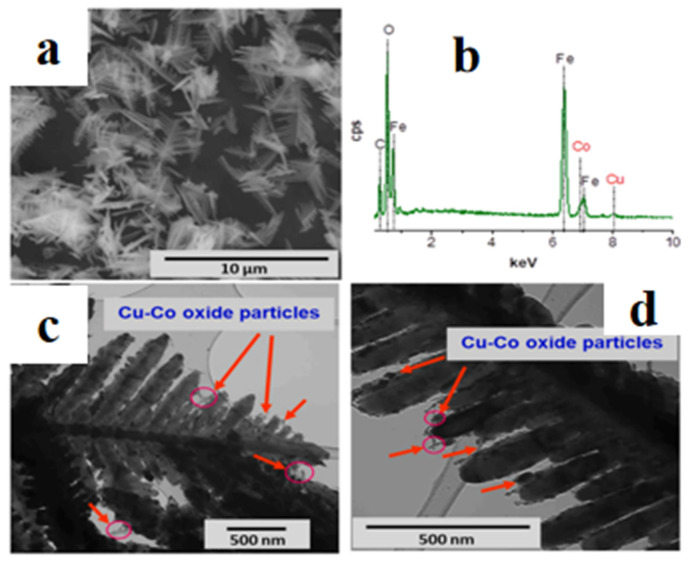
SEM and TEM analyses of the catalyst Cu-Co/α-Fe_2_O_3_: (**a**) SEM image of the particles; (**b**) EDX analysis; (**c**,**d**) TEM images showing Cu-Co oxide nanoparticles anchored on the α-Fe_2_O_3_ surface [14].

In addition to MOF-based templates, researchers have explored the design of hierarchical supports to anchor Cu–Co species to further enhance structural stability. Garzón-Cucaita et al. [14] developed a heterogeneous catalyst by supporting Cu–Co mixed oxides and silver on multi-branched α-Fe_2_O_3_ with a unique fern-leaf morphology (Figure 9). The high fractal dimension and roughness of the hematite support favor the uniform deposition of Cu–Co particles, which serve as the primary active phases for the degradation of Reactive Yellow 145 azo dye. The study emphasized that the synergistic interaction between the multi-branched support and the bimetallic oxides promotes a more efficient radical generation pathway. This branched architecture not only stabilizes the active metals but also enhances the accessibility of reactants to the catalytic centers, demonstrating the importance of morphology-controlled supports in boosting the performance of bimetallic catalysts (Figure 10).

### 3.3. Multi-Component Alloy Systems

#### 3.3.1. Ternary Alloy Systems

Ternary alloy systems represent a cutting-edge advancement in the field of heterogeneous Fenton-like catalysis [100]. By introducing a third metallic component, the electronic structure and coordination environment of the active sites can be further tuned. Compared to bimetallic catalysts, ternary alloys offer more diversified electron transfer pathways, effectively overcoming the slow reduction of high-valence metal ions (e.g., the reduction of Fe^3+^ to Fe^2+^) and improving the utilization efficiency of the oxidant [38].

**Figure 11:**
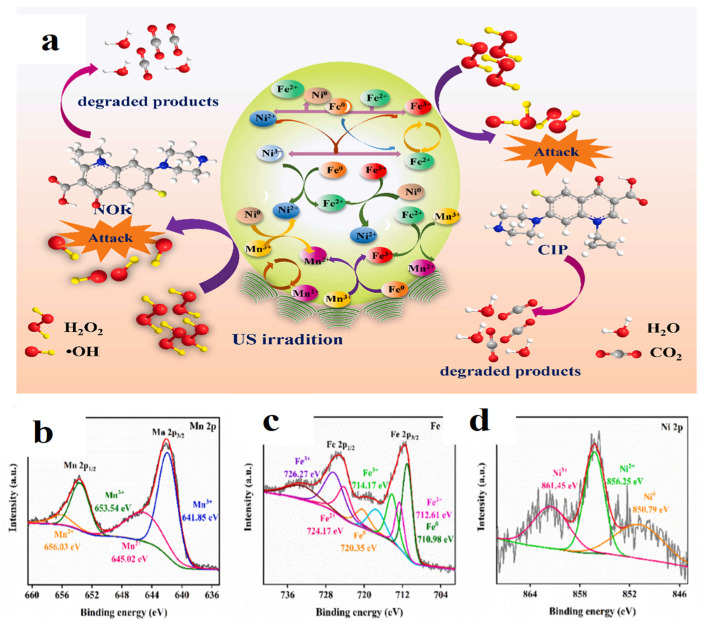
(**a**) Possible degradation mechanism of the heterogeneous sono-Fenton-like reaction based on Mn_3_O_4_−TA@FeNiB; XPS spectra of Mn_3_O_4_−TA@FeNiB composites [101]: Mn 2p (**b**) [101], Fe 2p (**c**) [101] and Ni 2p (**d**) [101].

Due to its high activity and low toxicity, iron (Fe) remains the core element in most ternary Fenton-like catalysts. Dang et al. [101] synthesized an amorphous FeNiB alloy supported on tannic acid-functionalized Mn_3_O_4_ (Mn_3_O_4_-TA@FeNiB) for the sono-Fenton-like degradation of antibiotics. In this system, the corrosion of the FeNiB alloy continuously releases Fe^2+^ and Ni^2+^, while the Mn^2+^/Mn^3+^ redox pair on the support provides additional active sites. This multi-metal synergy establishes a robust electron transfer network, significantly accelerating radical generation under ultrasonic irradiation (Figure 11).

Furthermore, Suligoj et al. [37] developed silica-supported Cu-Mn-Fe multi-component catalysts for solar-driven water treatment. The study revealed that while the Fe_3_O_4_ core provides magnetic properties for easy recovery, the catalytic activity is primarily driven by the synergistic disproportionation of H_2_O_2_ between Cu^2+^ species and isolated Mn cations embedded within the silica framework. Similarly, Song et al. [102] prepared an Fe/Mn/Cu/tourmaline catalyst, achieving an 86.79% mineralization rate of organophosphorus scale inhibitors through the coordination of three transition metals on the mineral support.

**Figure 12:**
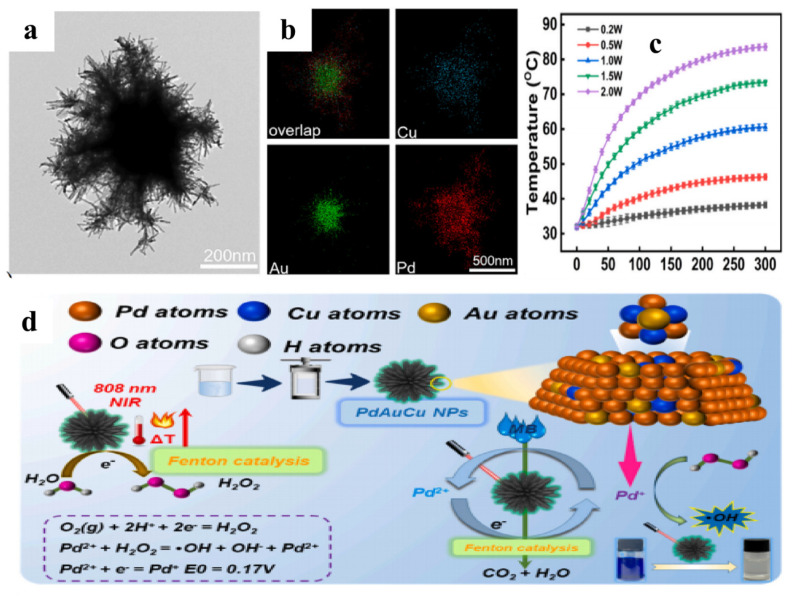
(**a**) TEM image of Pd−Au−Cu NPs [103]; (**b**) EDX mapping images of Pd−Au−Cu NPs [103]; (**c**) Temperature increase of Pd−Au−-Cu NPs based on concentration under near-infrared light [103]; (**d**) Synthesis of Pd−Au−Cu nanoparticles and photothermal catalytic process [103].

Integrating noble metals (such as Pd, Pt, or Au) into ternary alloys can introduce unique physical effects, such as Surface Plasmon Resonance (SPR) and photothermal conversion. He et al. [103] prepared coral-like Pd–Au–Cu trimetallic alloy nanoparticles for photothermally enhanced Fenton catalysis (Figure 12a). The presence of Au and Cu modulates the d-band center of Pd (Figure 12b), optimizing the adsorption energy of reactants. Under 808 nm near-infrared (NIR) laser irradiation, the alloy efficiently converts light energy into localized thermal energy (Figure 12c), providing the necessary activation energy for the rapid decomposition of H_2_O_2_ at Pd and Cu sites (Figure 12d).

**Figure 13:**
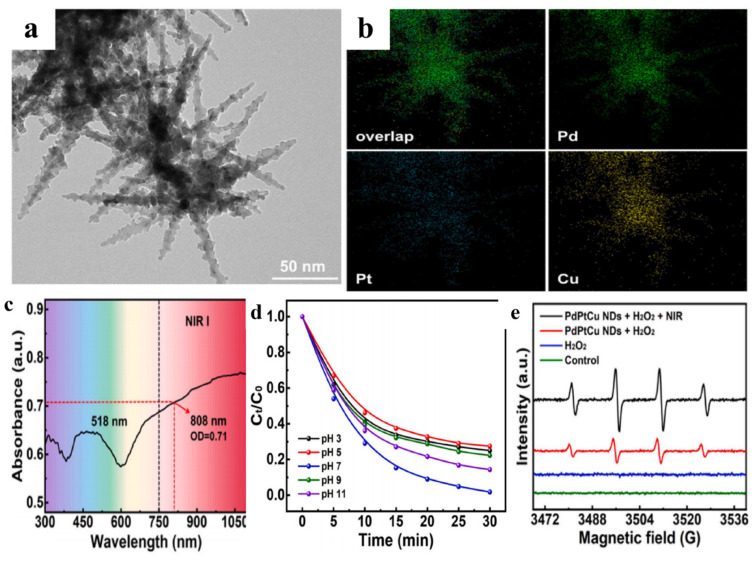
(**a**) TEM images of PdPtCu NDs [8]; (**b**) EDX mapping images of PdPtCu NDs; (**c**) UV-Vis characterization of PdPtCu NDs [8]; (**d**) Effect of pH on MB degradation (concentration of MB: 0.1 mg·mL^−1^, PdPtCu NDs:0.5 mg·mL^−1^ and H_2_O_2_: 5%, temperature: 50 °C) [8]; (**e**) DMPO-·OH spectrum of EPR [8].

Following a similar strategy, Zhang et al. [8] synthesized nanodendritic PdPtCu alloys. These ternary nanodendritic structures utilize the SPR effects of Pt and Pd to capture NIR light, while Cu atoms serve as the primary catalytic centers for hydroxyl radical (·OH) generation. The intimate atomic integration within the PdPtCu lattice ensures efficient interfacial electron transfer, thereby overcoming the limitations of traditional iron-based Fenton systems in neutral environments (Figure 13).

#### 3.3.2. High-Entropy Alloy (HEA) Systems

High-entropy alloys (HEAs), typically composed of five or more metallic elements in equiatomic or near-equiatomic proportions, have emerged as a transformative class of catalysts for Fenton-like reactions [104,105,106,107]. These materials are characterized by four core effects: the high-entropy effect, lattice distortion effect, sluggish diffusion effect, and the “cocktail” effect [108]. Their inherent structural disorder, combined with the synergistic interactions among multiple active sites, endows HEAs with exceptional catalytic activity and stability that often surpass those of traditional monometallic or bimetallic catalysts.

**Figure 14:**
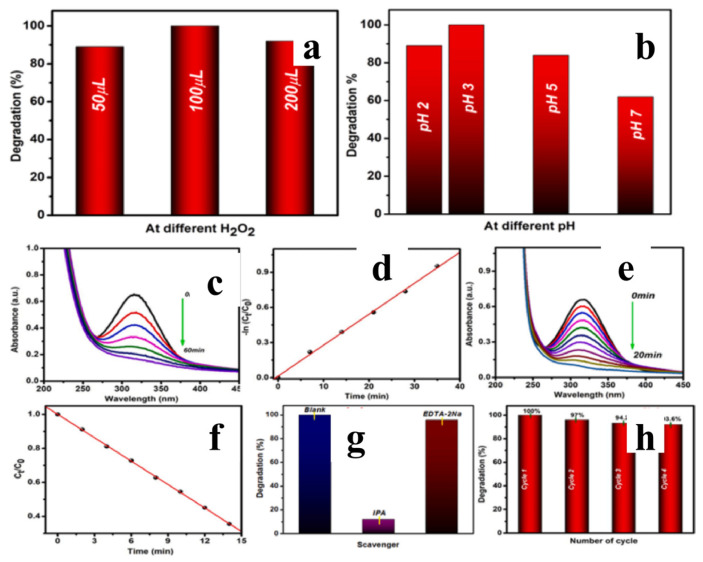
Photo-Fenton reaction of PNP (**a**) at different H_2_O_2_ amount [109] (**b**) at different pH value [109]. (**c**) UV−visible absorbance spectra of PNP degradation under Fenton-like conditions (in dark) [109] and (**d**) Kinetics of Fenton reaction (in dark) [109]. (**e**) UV−visible absorbance spectra for photo−Fenton PNP degradation (under visible light irradiation) [109]. (**f**) Kinetics of photo−Fenton degradation [109]. (**g**) Scavenger experiments for photo−Fenton degradation of PNP. (**h**) Recyclability of HEA sample [109].

In Fenton and photo-Fenton systems, HEAs exhibit a unique ability to facilitate multi-valence metal redox cycles. For instance, Anuraag et al. [109] successfully synthesized an MnFeNiCuBi high-entropy alloy with a face-centered cubic (fcc) structure via high-energy ball milling. Serving as an efficient catalyst for the decomposition of p-nitrophenol (PNP), the presence of multiple metallic elements on the alloy surface promotes the formation of a multi-component oxide shell. This generates a narrow bandgap of 1.44 eV, resulting in significant photo-Fenton activity under visible light. In the dark Fenton process, the catalyst achieved complete degradation of PNP within 60 min, whereas the photo-Fenton process required only 20 min. The typical effective temperature range for the MnFeNiCuBi high-entropy alloy (HEA) catalyzed Fenton-like reaction is 25–55 °C, with an apparent activation energy (Ea) as low as 12–25 kJ/mol, representing one of the lowest activation energies reported among multimetallic alloy systems. According to the Arrhenius equation, this ultra-low activation energy originates from the lattice distortion induced by the high-entropy effect and the multimetallic “cocktail effect.” The multivalent cycling of Fe/Ni/Mn/Cu/Bi establishes a multidimensional electron transfer network, further lowering the energy barrier for H_2_O_2_ activation. Consequently, complete degradation of p-nitrophenol (PNP) is achieved within 60 min under dark Fenton conditions (25 °C) and in only 20 min under photo-Fenton conditions (25 °C). Even as the temperature increases to 55 °C, the catalyst exhibits no significant deactivation (Figure 14).

**Figure 15:**
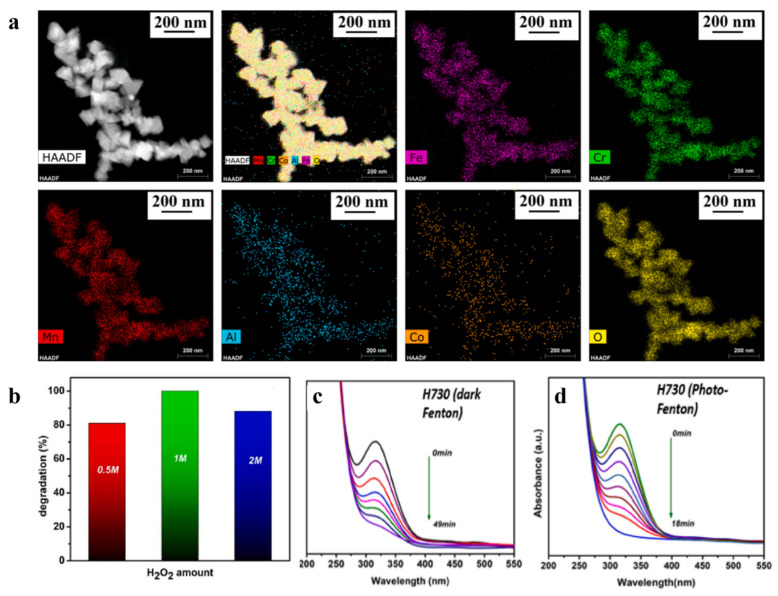
(**a**) HAADF-STEM-EDS elemental mapping for Fe, Cr, Mn, Al, Co and O present in H730 sample; (**b**) Fenton-like degradation under the H730 catalytic systems [110]; (**c**) Fenton degradation on H730; (**d**) photo-Fenton degradation on H730.

Furthermore, the synergistic effect produced by the compositing of reduced graphene oxide (r-GO) supports with high-entropy alloys further optimizes their Fenton-like catalytic activity. Kumar et al. [110] prepared r-GO-coated magnetic high-entropy alloy/oxide nanocomposites (r-GO@MHEA/O) and applied them to Fenton-like and photo-Fenton catalytic reactions. The synergy formed by the various metallic elements in this HEA system (such as Fe, Co, Ni, Cu, and Zn) effectively facilitates valence state cycling among metal species (e.g., Fe^3+^/Fe^2+^, Co^3+^/Co^2+^, Cu^2+^/Cu^0^). This significantly accelerates the electron transfer process, thereby enhancing the generation efficiency of reactive oxygen species (ROS) such as ·OH, leading to a dramatic improvement in catalytic degradation performance (Figure 15).

The synergistic effect of multi-metal active sites manifests in high intrinsic activity and atomic utilization efficiency. Their unique electronic structure can enhance catalytic performance by modulating the adsorption energy between reactants and the catalyst surface. Yao et al. [111] successfully prepared nitrogen-doped carbon-supported Cu_12_Pd_11_Fe_10_Co_11_Ni_12_ high-entropy alloy nanoparticles (HEA-NPs) using a one-pot oil-phase synthesis followed by a controlled pyrolysis strategy. In an HEAs-PMS system utilized for peroxymonosulfate (PMS) activation in a Fenton-like reaction, the phenol removal rate reached 100% within 10 min. This system transforms phenolic pollutants into high-molecular-weight polymer products through a unique non-mineralization pathway, demonstrating extremely high electron utilization efficiency and significantly reducing oxidant consumption.

## 4. Performance Enhancement Strategies for Alloy-Based Fenton-like Catalysts

### 4.1. Co-Catalyst Strategy

The co-catalysis strategy enhances the performance of Fenton-like systems by introducing a second component as either a co-catalyst or an electronic regulator [112,113,114,115,116,117]. For instance, the introduction of quinone compounds or graphene as a second component can promote the redox cycle of metal active centers and accelerate active site regeneration due to their electron-rich characteristics [118,119,120,121,122,123]. By modulating electron transfer, stabilizing active sites, and intensifying synergistic effects, the co-catalyst strategy provides diversified pathways for optimizing the performance of multi-metal alloy catalysts [124,125,126].

Yang et al. [112] demonstrated that micro-alloying iron-based metallic glasses with trace amounts of nitrogen significantly improves catalytic efficiency. The nitrogen atoms effectively modulate the electronic structure of the Fe_78_Si_9_B_13_ alloy, inhibiting the formation of surface oxides and ensuring the continuous exposure of a large number of Fe^0^ active sites. Furthermore, the synergy between different metallic species within an alloy can be regarded as a mutual co-catalytic process. For example, the coexistence of Cu and Co species in Cu-Co binary metal–organic framework (MOF) nanosheets promotes more efficient electron transfer compared to their monometallic counterparts, resulting in superior oxidative activity for the degradation of norfloxacin. Beyond traditional metals, Song et al. [102] utilized tourmaline as a promoting component in an Fe/Mn/Cu catalyst. The self-polarization effect provided by tourmaline enhanced the degradation of organophosphorus scale inhibitors, achieving a mineralization rate of 86.79% for 1-hydroxyethylidene-1,1-diphosphonic acid (HEDP).

### 4.2. External Field-Assisted Strategies

External field-assisted Fenton technology aims to enhance reaction efficiency and overcome traditional application limitations by introducing external energy fields such as light, electricity, ultrasound, and heat. Currently, several significant research directions have emerged [127,128,129,130,131,132]. Among these, photo-Fenton and electro-Fenton technologies have advanced most rapidly. Light assistance facilitates the separation of photogenerated electron-hole pairs in catalysts, enhancing the iron cycle and the in situ generation of hydrogen peroxide (H_2_O_2_) [133]. Electrical assistance achieves efficient regeneration of iron ions via cathodic reduction and enables the in situ electrochemical production of reactive oxygen species (ROS) [134,135]. Furthermore, ultrasound assistance strengthens mass transfer and refreshes reaction interfaces through cavitation effects, while thermal assistance accelerates reaction kinetics [136,137,138,139,140,141].

The ultrasonic field utilizes the localized high temperatures, high pressures, and micro-jets generated by acoustic cavitation to effectively eliminate mass transfer resistance in heterogeneous reactions and activate catalytic sites. Zhang et al. [142] synthesized Cu or Fe-doped NaP zeolites (NaPultm) using an ultrasound-assisted hydrothermal method. Their study demonstrated that under an ultrasonic power intensity of 30% and a density of 60 mL/W, acoustic cavitation not only significantly shortened the crystallization time of the catalyst but also induced the efficient distribution of active metals onto the zeolite framework. This led to a dramatic enhancement in the oxidative decolorization efficiency of the system for methylene blue (MB) and methyl violet (MV-6B).

Under ultrasound assistance, the effective temperature range for the Fenton-like reaction catalyzed by the Cu/Fe-doped NaP zeolite catalyst is reduced to 25–30 °C, with the apparent activation energy (Ea) decreasing from 28 kJ/mol (without ultrasound) to 10–20 kJ/mol. The cavitation effect of ultrasound generates localized micro-jets that continuously refresh the reaction interface at the catalyst surface, thereby lowering the adsorption energy barrier for H_2_O_2_ at the active sites. Based on the Arrhenius equation, this substantial reduction in Ea leads to a 3–4-fold increase in the reaction rate constant at 25 °C, resulting in the Methylene Blue (MB) decolorization efficiency rising from 80% to 99%.

Thermal assistance strategies lower the reaction activation energy barrier by increasing the thermal energy of the reaction system. Fashi et al. [125] conducted a comparative study in a continuous flow system regarding the enhancement effects of heat treatment versus UVC irradiation on the oxidation of corn starch by a CuSO_4_/H_2_O_2_ system. Experimental data confirmed that under a thermal assistance condition of 50 °C, the system exhibited oxidative performance comparable to that of UV-light assistance. The thermal input accelerated the kinetics of Cu(II) conversion to Cu(I) and promoted H_2_O_2_ decomposition, providing a green and efficient process scheme for the large-scale continuous degradation of macromolecular organic matter.

Integrating external physical fields, such as electric fields or light energy, can circumvent the thermodynamic or kinetic limitations of traditional Fenton-like reactions. Among these, photothermal enhancement has become a highly promising direction. He et al. [103] synthesized coral-like Pd-Au-Cu trimetallic alloy nanoparticles, which exhibited a strong photothermal effect under near-infrared (NIR) laser irradiation. This localized heating accelerated the Fenton-like catalytic process, significantly increasing the removal rate of organic dye pollutants.

Electrochemical assistance is another widely explored field, particularly through heterogeneous electro-Fenton (HEF) processes. Yin et al. [124] emphasized that the efficiency of electro-Fenton (EF) technology depends on the selective two-electron oxygen reduction reaction for the in situ generation of H_2_O_2_ and the subsequent efficiency of its conversion into ·OH. Liu et al. [9] successfully prepared metal–organic framework (MOF)-derived Fe/Ni@C marigold-like nanosheets and utilized them as high-performance HEF cathodes. The bimetallic Fe/Ni sites synergistically catalyzed the activation of in situ-generated H_2_O_2_, achieving the efficient degradation of oxytetracycline. Similarly, Cui et al. [143] developed a self-supporting Fe/Co bimetallic nitrogen-doped porous carbon (Fe/Co-NPC) electrode. This integrated structure facilitated rapid Rhodamine B removal across a wide pH range (3.0–9.0) by promoting efficient interfacial electron transfer and high H_2_O_2_ yields.

### 4.3. Supported Composite Material Strategy

The construction of supported composite materials effectively addresses the issues of metal leaching and catalyst agglomeration while imparting additional functional properties to the materials [116,144,145,146,147,148,149,150,151,152]. Carbon-based materials have become the most commonly utilized supports due to their high electrical conductivity and large specific surface area [153,154,155]. As demonstrated by Liu et al. [9] the carbon matrix in Fe/Ni@C nanosheets not only stabilizes the bimetallic active sites but also enhances the conductivity required for electrocatalysis. Cui et al. [143] further showed that nitrogen-doped porous carbon networks derived from metal–organic frameworks (MOFs) provide abundant defect sites, thereby improving the accessibility of reactants to Fe and Co active centers [156].

Natural minerals also serve as excellent supports for multi-metal catalysts. Song et al. [102] employed tourmaline as a support for Fe/Mn/Cu catalysts. This support facilitates the recovery of inorganic phosphorus generated during the mineralization of organic pollutants, achieving a recovery rate of 93.71%. Furthermore, the morphology of the composite material plays a critical role; Zhou et al. emphasized that the nanosheet structure of Cu–Co MOFs provides a vast active surface area, which is conducive to the adsorption and subsequent degradation of macromolecular organic matter, such as norfloxacin [35].

## 5. Summary and Future Perspectives

### 5.1. Summary

Multi-metal alloy catalytic materials have provided critical support for the breakthrough development of Fenton-like technologies, owing to their significant advantages in three dimensions: synergistic effects, structural stability, and broad pH adaptability. Synergistic effects, driven by electronic coupling and interactions between multiple metals, optimize the electronic structure of active sites and accelerate redox cycles. This not only overcomes the inherent limitations of monometallic catalysts but also leverages the “cocktail effect” of high-entropy alloys to achieve fine-tuning of active sites, pushing catalytic performance to new heights. Structural stability, maintained through mechanisms such as lattice distortion and surface regulation, effectively inhibits metal leaching and active site agglomeration, ensuring long-term recyclability. This stability directly supports broad pH adaptability, allowing the catalytic system to maintain high activity in neutral to weakly alkaline environments, thereby significantly reducing the costs associated with pH adjustment in practical applications. Furthermore, the performance of multi-metal alloys can be further optimized through strategies such as co-catalyst synergy, external field assistance, and supported composite modification. These approaches enhance the activity, stability, and environmental adaptability of catalysts across multiple dimensions, driving their evolution toward high efficiency and multifunctionality and offering more possibilities for practical applications in the field of water treatment.

### 5.2. Challenges and Future Outlook

The engineering application of multi-metal alloy Fenton-like catalysts currently faces four primary challenges. Regarding large-scale synthesis, maintaining the uniform distribution of multiple elements at the atomic level (particularly for five or more metals in high-entropy alloys) remains a technical bottleneck. Some high-performance materials, such as MOF-derived structures, involve complex preparation processes and high costs, making cost-effective mass production difficult. At the mechanistic level, the understanding of dynamic processes at multi-metal interfaces remains insufficient. The redox cycles of multi-valent metals, electron transfer pathways, and synergistic mechanisms lack in-depth elucidation through in situ characterization and systematic theory. In terms of practical wastewater adaptability, anions such as Cl^−^ and HCO_3_^−^ in complex matrices tend to quench reactive species, while coexisting pollutants interfere with catalytic selectivity. The stability and reliability of catalysts in real water bodies urgently require validation. Finally, technological economics must be optimized; current research focuses predominantly on performance enhancement, lacking systematic Life Cycle Assessments (LCA) and cost–benefit analyses. Furthermore, as some catalysts rely on scarce metals, the efficient utilization of high-abundance metals requires further exploration.

Future research should advance through the synergy of material design, mechanistic analysis, system integration, and economic optimization to accelerate the transition from laboratory research to engineering application. At the material and system level, “smart” catalysts with environmental responsiveness should be developed and coupled with external fields (light, electricity, heat) and renewable energy sources to construct efficient, low-energy integrated treatment systems. Mechanistic studies must integrate in situ characterization with theoretical calculations to deeply reveal interfacial electron transfer, the dynamic evolution of active sites, and synergistic mechanisms, thereby guiding the precision design of catalysts. For engineering applications, efforts should focus on developing robust catalysts resistant to interference from complex water matrices and enhancing their adaptability and long-term stability in actual wastewater through loading and compositing strategies. Concurrently, it is essential to systematically conduct techno-economic analyses and Life Cycle Assessments to optimize preparation processes, promote the application of high-abundance, low-cost metals, and facilitate the efficient, green, and large-scale application of multi-metal alloy catalysts.

## Figures and Tables

**Table 1:** Reaction Equations and Activation Energies of Metal-based Fenton reactions.

System	Reaction Equation	Eₐ/(kJ·mol^−1^)	Reference
Fe^2+^/H_2_O_2_	${\mathrm{Fe}}^{2+}+H_{2}O_{2}\;\to {\;\mathrm{Fe}}^{3+}+\cdot \mathrm{OH}+{\mathrm{OH}}^{-}$	39.3	[26]
Cu^+^/H_2_O_2_	${\mathrm{Cu}}^{+}+H_{2}O_{2}\;\to \;{\mathrm{Cu}}^{2+}+\cdot \mathrm{OH}+{\mathrm{OH}}^{-}$	40~60	[27]
Co^2^/H_2_O_2_	${\mathrm{Co}}^{2+}+H_{2}O_{2}\;\to \;{\mathrm{Co}}^{3+}+\cdot \mathrm{OH}+{\mathrm{OH}}^{-}$	50~70	[28]
Mn^2+^/H_2_O_2_	${\mathrm{Mn}}^{2+}+{\;H}_{2}O_{2}\;\to \;{\mathrm{Mn}}^{3+}+\cdot \mathrm{OH}+{\mathrm{OH}}^{-}$	60~80	[29]
H_2_O_2_	$2H_{2}O_{2}\;\to \;2H_{2}O+{\;O}_{2}$	226	[24]
Fe^2+^/PDS	${\mathrm{Fe}}^{2+}+S_{2}O_{8}^{2-}\;\to \;{\mathrm{Fe}}^{3+}+{\mathrm{SO}}_{4}^{2-}+{\mathrm{SO}}_{4}^{\cdot -}$	50.2	[30]
Cu^+^/PDS	${\mathrm{Cu}}^{+}+S_{2}O_{8}^{2-}\;\to \;{\mathrm{Cu}}^{2+}+{\mathrm{SO}}_{4}^{2-}+{\;\mathrm{SO}}_{4}^{\cdot -}$	45~55	[31]
PDS	$S_{2}O_{8}^{2-}\;\to \;2{\mathrm{SO}}_{4}^{\cdot -}$	100~140	[32]
CO_2_^+^/PMS	${\mathrm{Co}}^{2+}+{\;\mathrm{HSO}}_{5}^{-}\;\to \;{\mathrm{Co}}^{3+}+{\;\mathrm{SO}}_{4}^{\cdot -}+{\mathrm{OH}}^{-}$	30~40	[28]
Fe^2+^/PMS	${\mathrm{Fe}}^{2+}+{\mathrm{HSO}}_{5}^{-}\;\to \;{\mathrm{Fe}}^{3+}+{\;\mathrm{SO}}_{4}^{\cdot -}+{\mathrm{OH}}^{-}$	40~50	[33]
PMS	$2{\mathrm{HSO}}_{5}^{-}\;\to \;2{\mathrm{SO}}_{4}^{2-}+O_{2}+2H^{+}$	140~160	[34]

**Table 2:** Common heterogeneous metal species applied in Fenton and Fenton-like processes (Heterogeneous metal).

Metal	Reactants/Contaminants	Reaction Condition	Efficiency	Reference
Fe	H_2_O_2_/Reactive Black5 (RB5)	Catalyst 1 g/L, H_2_O_2_ 1 mM, RB5 100 mg/L, pH 3.0–6.5	85%	[42]
	NH_2_-MIL101(Fe,Cu)/WO_3_/H_2_O_2_/(LEV)	Visible light, H_2_O_2_ dosage optimized	90%	[43]
	H_2_O_2_/RhodamineB (RhB)	Fe_3_Cu_2_/HZSM–5 catalyst, room temperature	100%	[44]
Cu	H_2_O_2_/Organic Dyes	Nanodendritic alloy, Surface plasmon resonance-driven	80%	[8]
	H_2_O_2_/Doxycycline	Cu–Ni LDH decorated biochar, pH 8.0	99.9%	[7]
Co	H_2_O_2_/Norfloxacin (NOR)	Cu–Co MOF nanosheets, bimetallic synergistic effect	95.37%	[35]
	H_2_O_2_/Reactive Yellow145 (RY145)	Cu–Co/α–Fe_2_O_3_ multibranched catalyst	80%	[14]

## Data Availability

No new data were created or analyzed in this study. Data sharing is not applicable to this article.

## Abbreviations

The following abbreviations are used in this manuscript:

AOPs	Advanced Oxidation Processes
·OH	hydroxyl radicals
PDS	persulfate
PMS	peroxymonosulfate
ROS	reactive oxygen species
ZVI	zero-valent iron
EXAFS	Extended X-ray Absorption Fine Structure
XANES	X-ray Absorption Near Edge Structure
HEAs	high-entropy alloys
r-GO	reduced graphene oxide
RhB	Rhodamine B
SMX	sulfamethoxazole
MB	methylene blue
EDTA	ethylenediaminetetraacetic acid
